# β1 Integrins as Therapeutic Targets to Disrupt Hallmarks of Cancer

**DOI:** 10.3389/fphar.2015.00279

**Published:** 2015-11-24

**Authors:** Anne-Florence Blandin, Guillaume Renner, Maxime Lehmann, Isabelle Lelong-Rebel, Sophie Martin, Monique Dontenwill

**Affiliations:** Department “Tumoral Signaling and Therapeutic Targets,” Faculty of Pharmacy, UMR7213 Centre National de la Recherche Scientifique, University of StrasbourgIllkirch, France

**Keywords:** integrins, hallmarks of cancer, proliferation, migrationinvasion, resistance to cell death, angiogenesis, therapeutic target

## Abstract

Integrins belong to a large family of αβ heterodimeric transmembrane proteins first recognized as adhesion molecules that bind to dedicated elements of the extracellular matrix and also to other surrounding cells. As important sensors of the cell microenvironment, they regulate numerous signaling pathways in response to structural variations of the extracellular matrix. Biochemical and biomechanical cues provided by this matrix and transmitted to cells via integrins are critically modified in tumoral settings. Integrins repertoire are subjected to expression level modifications, in tumor cells, and in surrounding cancer-associated cells, implicated in tumor initiation and progression as well. As critical players in numerous cancer hallmarks, defined by Hanahan and Weinberg (2011), integrins represent pertinent therapeutic targets. We will briefly summarize here our current knowledge about integrin implications in those different hallmarks focusing primarily on β1 integrins.

## Introduction

In the setting of cancer, six hallmarks enabling a cell to become tumorigenic and ultimately malignant have been defined by Hanahan and Weinberg (2000). As such cancer cells have the abilities to sustain proliferative signaling, to evade growth suppressors, to resist to cell death, to enable replicative immortality, to induce angiogenesis and to activate invasion and metastasis. This list has been extended recently by the authors taking into account the new progresses made in the past decade with the proposal of two new hallmarks comprising the reprogramming of energy metabolism and the evasion of immune destruction (Hanahan and Weinberg, 2011). A recent review emphasized the modulation of these hallmarks by the extracellular matrix (Pickup et al., 2014). Integrins belong to one of the most studied family of matricellular receptors. These heterodimeric αβ cell surface receptors sense the extracellular matrix with high flexibility (Hanein and Horwitz, 2012; Hohenester, 2014) triggering thereby specific answers in both physiological and pathophysiological conditions. In humans, 18 α and 8 β subunits have been characterized enabling about 24 heterodimeric combinations. The Arg-Gly-Asp (RGD) binding integrins belong to the most studied subfamily including αvβ1, αvβ3, αvβ5, αvβ6, αvβ8, α5β1, and αIIbβ3. The RGD motif is available in ECM components such as fibronectin, vitronectin, osteopontin, and fibrinogen. Cell binding to collagen or laminin involves either β1 (α1β1, α2β1, α10β1, α11β1, α6β1…) or β4 (α6β4…) subunits-containing integrins. Integrins signaling operates through the integrin adhesome which appears complex (Winograd-Katz et al., 2014). Interactions of integrins with soluble or membrane-localized elements as well as with cytoplasmic adaptors or catalytic partners (kinases, phosphatases, proteases …) define series of coordinated and spatiotemporal regulated processes. Mechanistic insights into the fine tuning of integrin signaling thereby revealing the high versatility of the cell answer to integrin-driven stimuli were enabled by the development of new technologies (Rossier et al., 2012; Robertson et al., 2015). Expression and activity of integrins and of their adhesome components have been implicated in various diseases including cancer. Integrins are well recognized as valuable tumor therapeutic targets although essentially in preclinical studies (Desgrosellier and Cheresh, 2010; Schaffner et al., 2013). A recent review emphasized their capability to regulate cancer stemness, metastasis, and drug resistance (Seguin et al., 2015). They remarkably impact on the hallmarks of cancer as defined above. In this review, our aim is to update the knowledges with the most recent data in the field focusing particularly on β1 integrins and their roles in the tumor progression. Integrins containing the β1 subunit constitute the largest subgroup and appear overexpressed in several solid tumors compared to control tissues (Paulus et al., 1993; Barkan and Chambers, 2011; Fabricius et al., 2011; Lahlou and Muller, 2011; Schaffner et al., 2013).

## Integrins and a sustained proliferative signaling

Integrins contribute to the cell cycle progression in physiological and pathological situations (reviewed in Moreno-Layseca and Streuli, 2014). The cross-talk between integrins and growth factor receptors (GFR) is well established especially in sustaining the cell proliferative signaling. Several GFR are concerned of which the epidermal growth factor receptor (EGFR), the hepatocyte growth factor receptor (HGFR/cMet), the platelet-derived growth factor receptor (PDGFR) and the vascular endothelial growth factor receptor (VEGFR). Direct activation of GFR by integrins was first described in normal cells. In endothelial cells, integrins phosphorylate EGFR even in the absence of EGF (Moro et al., 2002). More recently, β1 integrin downregulation decreased the phosphorylation of c-Met and of EGFR in hepatocytes during liver regeneration (Speicher et al., 2014). Keratinocytes stimulation by EGF modulates constituents of focal adhesion complexes including β1/β3 integrins and FAK (Eberwein et al., 2015). The synergistic relationship between integrins and GFR is also highlighted in tumor progression (Ivaska and Heino, 2011). Physical interactions between integrins and GFR have been demonstrated by co-immunoprecipitation experiments (α5 integrin and EGFR; Morozevich et al., 2012) or by FRET analysis on patient tumor slices (β1 integrin and ERBB1; Petras et al., 2013). Interestingly, proliferative cooperation between ECM receptors and GFR may also be achieved through direct ECM-GF interactions (Vlahakis et al., 2005; Oommen et al., 2011; Dong et al., 2014; Han et al., 2014; Zhu and Clark, 2014). The fine molecular tuning of the integrin-GFR interplay implicates other partners either from the ECM or from the cellular compartment. The matricellular protein CCN1 (CYR61/CCN1, cysteine-rich protein 61) inhibits EGFR-dependent hepatocytes proliferation through ROS accumulation induced by α6β1 integrin in liver carcinoma (Chen et al., 2015). Tenascin-C induces a physical association of PDGFR- β and α5β1 integrin resulting in prolonged activation of PDGFR- β and deregulated proliferation of fibroblast cell line (Tanaka et al., 2014). EGFR signaling regulates ILK (Integrin Linked Kinase) to increase gastric cancer cells proliferation (Tseng et al., 2014). In epidermoid carcinoma cells, EGF stimulation modulates α5β1 activation state by phosphorylation of Filamin-A (Vial and McKeown-Longo, 2012). In the same model, α5β1 integrin inhibition reduces EGFR phosphorylation implicated in cell proliferation (Morozevich et al., 2012). Scaffolding proteins such as tensin4 (TNS4) may create a functional complex between cMet and integrin β1 (Muharram et al., 2014). Hepatocellular carcinoma progression has been blocked by nanoparticle-formulated siRNA targeting β1 and αv integrins through reduced activation of MET oncogene (Bogorad et al., 2014). Integrins and GFR thus mainly interact through cross-regulated signaling pathways. In the case of EGFR and α5β1integrin, common way of intracellular trafficking may also potentiate their functions (Caswell et al., 2008). The overexpression of GFR and/or the expression of constitutively active mutants (such as the EGFRvIII mutant; Guo et al., 2015) are hallmarks of different tumor types and boost the proliferation of tumoral cells. Powerful therapeutic strategies may thus include simultaneous integrin/GFR targeting for selected tumors and patients (Eke et al., 2015).

## Integrins and the evasion of growth suppressors and the resistance to cell death

### Evading growth suppressor

p53 one of the most prominent tumor suppressor, is mutated in about 50% of cancers (Ciriello et al., 2013). Wild type p53 signaling is nevertheless altered in a large majority of tumors by alternative pathways such as deletions/mutations of endogenous activators or amplifications of inhibitors (Brennan et al., 2013). Integrin signaling may be added to the list of p53 activity regulators. We have shown that α5β1 integrin impairs the p53 activation by chemotherapeutic drugs (Martinkova et al., 2010; Janouskova et al., 2012, 2013; Martin et al., 2012). Similar results obtained by others in breast carcinoma cells showed enhanced expression of p53 upon depletion of α2β1 integrin (Morozevich et al., 2015). In glioblastoma, an overexpression of α5 integrin was recorded in p53 wild type tumors (Janouskova et al., 2012) explaining their resistance to therapies. Conversely, in ovarian tumors with a mutated p53 overexpression of the β4 integrin leads to a metastasis advantage (Lee et al., 2015). In squamous cell carcinoma, cooperation between p53 and αv integrin, impacts on tumor induction and growth (Savar et al., 2015). Integrin signaling pathways have been highlighted in the regulation of p53 activity. Our recent data demonstrated participation of the integrin/AKT/PEA15/caspase8 axis in the inhibition of p53 (Renner et al., 2015). As reported by others, the integrin-downstream kinase FAK, has the capability to inhibit p53 through direct physical interaction in the nucleus or cytoplasm thus linking signaling from the ECM to the nucleus (Golubovskaya and Cance, 2011; Golubovskaya, 2014). Interestingly, a regulatory loop exists between FAK and p53 (Golubovskaya et al., 2008) similar to the one we described between α5 integrin and p53 (Renner et al., 2015). Finally, abrogation of α5 integrin or FAK signaling concomitantly with activation of p53 leads to tumor cell apoptosis (Gillory et al., 2015; Renner et al., 2015). Reactivation of p53 appears as a pertinent strategy for numerous tumors (Selivanova, 2014) and, as suggested above, blocking either integrins or their downstream signaling pathways may offer new opportunities to synergistically enhance the p53 tumor suppressor effects.

### Resisting cell death

Maintenance of cell survival through ECM-integrin interactions has been recognized for a long time in development and in tissue homeostasis. Loss in cell adhesion will block the pro-survival integrin-dependent signaling pathways including PI3K/AKT, MEK/ERK, FAK, NFκB, and/or ILK leading to a particular form of apoptosis named anoikis (Griffiths et al., 2011; Vachon, 2011). Resistance to anoikis promotes tumor progression and favors emergence of metastasis (Paoli et al., 2013; Buchheit et al., 2014). The “integrin switch” includes changes in their expression profile and functionality during cell detachment from the ECM thus overcoming anoikis and allowing tumor cell survival and metastasis (Janes and Watt, 2004). New contributors to anoikis resistance through integrin pathway modulations were recently discovered. In melanoma cells, TIMP1, a member of the metalloproteinase inhibitors, was shown to form a complex with CD63 and integrin β1 conferring resistance to anoikis (Toricelli et al., 2013). Depletion of cytoplasmic FER, a non-receptor tyrosine kinase, by increasing the expression of α6β1 integrin decreased anoikis resistance in breast cancer cells (Ivanova et al., 2013). Vacuolar-ATPase inhibitor has been shown to reduce active β1 integrins and to regulate anoikis resistance in several cancer cells (Schempp et al., 2014). Zinc finger transcription factor ZNF304 transcriptionally regulates the β1 integrin and prevents anoikis (Aslan et al., 2015). The miR-26a targeting of α5 integrin promotes anoikis in human hepatocellular carcinoma (Zhang et al., 2015b). Finally, atypical anoikis involving necrosis and autophagy in glioma cells was induced by cilengitide, an αvβ3/β5 integrin inhibitor (Silginer et al., 2014). Very recently, suppression of anoikis was attributed to integrin endosomal signaling (Alanko et al., 2015). These recent examples document the different ways for a tumoral cell to engage for resisting to cell-detachment induced apoptosis by means of modulation of integrin expression and functions.

### Resistance to therapies as a consequence

As the therapeutic protocols aim to eradicate the tumors and avoid recurrences, the best strategy would be to induce cell death. As supported by their pro-survival capacities, integrins participate to the resistance toward therapies including radio-, chemo- and targeted therapies (Aoudjit and Vuori, 2012; Nistico et al., 2014; Shishido et al., 2014; Eke and Cordes, 2015; Naci et al., 2015).

Research from the group of Cordes largely confirmed that β1 integrins induce radioresistance in head and neck cancers (Eke et al., 2012, 2015; Dickreuter et al., 2015; Steglich et al., 2015) whereas similar results have been reported by others in breast cancer (Nam et al., 2009, 2013; Ahmed et al., 2013). Resistance to radiotherapy has also been linked to αvβ3/β5 integrins (Monferran et al., 2008; Skuli et al., 2009; Ning et al., 2010; Ducassou et al., 2013; Lanvin et al., 2013). β1 integrins also modulate solid tumor responses to chemotherapies (Howe and Addison, 2012; Sorensen et al., 2015). In glioblastoma, we demonstrated the crucial role of α5β1 integrin in the resistance to Temozolomide (Martinkova et al., 2010; Janouskova et al., 2012). IGFBP-2 was involved in this resistance (Holmes et al., 2012; Han et al., 2014). Chemoresistance against doxorubicin by means of α2β1 integrin activation was recently noted in leukemia (Naci et al., 2012). Interestingly, an anchorage-independent form of chemoresistance may exist in leukemia cells implicating only the α integrin subunit and its cytoplasmic tail sequence KXGFFKR (Liu et al., 2013). If confirmed in other tumors, this will constitute a new concept in the field of integrin-dependent chemoresistance.

Integrins are also coopted candidates for innate and acquired resistance provoking tumor recurrence. In melanoma, the mutant BRAF inhibitor, vemurafenib, drives an adhesion signaling network involving α5β1 integrin and implicated in the drug resistance (Fedorenko et al., 2015). BRAF inhibition also activated a β1 integrin/FAK signaling pathway in the fibroblastic tumor stroma promoting tumoral cell survival (Hirata et al., 2015). In breast cancer cells, acquired resistance to tamoxifen is mediated by cancer-associated fibroblast-derived fibronectin which induces β1 integrin-dependent signaling in adjacent tumoral cells (Yuan et al., 2015). Ovarian taxol-resistant tumor populations exhibit an increase in β1 integrin expression and microtubule dynamics (McGrail et al., 2015). One of the most studied resistance mechanism addresses the integrin-GFR crosstalk. The importance of αvβ3 integrin/KRAS axis in the resistance of various solid tumors toward EGFR targeted therapies has been demonstrated (Seguin et al., 2014). β1 integrin is also implicated in resistance to anti-EGFR therapies (Huang et al., 2011; Morello et al., 2011; Eke et al., 2013; Kanda et al., 2013). By contrast, a recent study showed that β1 integrin and EGFR inhibitions are inefficient for radio- and chemo-sensitization of colorectal carcinoma cell *in vitro* (Poschau et al., 2015). Cooperation between β1 integrin and c-Met regulates tyrosine kinase inhibitor resistance in lung cancer (Ju and Zhou, 2013).

In solid tumors, as resistance to therapies can be mediated by GFR and β1 integrin, targeting of β1 integrin simultaneously with GFR inhibitors may be a promising therapeutic approach. In addition, new data stress side-effects of targeted therapies on the tumor-surrounding microenvironment that may affect the integrin signaling pathways to reinforce their resistance mechanisms.

## Integrins and invasion/metastasis

Other key biological process of cancer progression comprises local invasion and metastatic dissemination of tumor cells which present interconnected pathways with resistance to therapies (Alexander and Friedl, 2012). Cell adhesion to ECM is central to the migration/invasion/metastasis process and implicates largely integrins (Scales and Parsons, 2011; Esposito and Kang, 2014; Naci et al., 2015). It is known for a long time that integrins regulate MMPs (matrix metalloproteinases) facilitating ECM degradation and remodeling. New data extend these findings (Borrirukwanit et al., 2014; Missan et al., 2015; Schlomann et al., 2015). New components are still being discovered contributing to the activity/function of integrins in cancer. Among those, actin-binding proteins or nucleation/assembly factors were recently reported to play crucial roles in the proinvasive activity of integrins. High expression of Profilin-1 (PFN1-a regulator of actin polymerization) was associated to tumor infiltration and lymph node metastasis. In gastric cancer, silencing PFN1 reduced β1 integrin expression and prevented FAK signaling (Cheng et al., 2015). Formin-like 2 (FMNL2—actin nucleation and assembly factor), upregulated in several metastatic cancers, interacts with RhoC to drive α2β1 and α5β1 integrin internalization/trafficking and invasive motility of cancer cells (Wang et al., 2015). Invasive migration of cancer cells into fibronectin-rich 3D ECM was reported to be enhanced following Rab-coupling protein (RCP)-driven endocytic recycling of α5β1 integrin. Invasive cells exhibit dynamic actin spike protrusions that are Arp2/3-independent but requires ROCK-mediated activation of FHOD3 (member of the formin family of protein; Paul et al., 2015). Integrin signaling can be rewired to increase tumor invasiveness during tumor metastasis by a novel mechanism recently described (Leyme et al., 2015). Integrins and G protein-coupled receptor traditionally trigger independent signaling but interestingly it was shown that integrin signaling requires the activation of the trimeric G protein Gαi by GIV or Girdin. In breast cancer cells, GIV colocalize with β1 integrin in invadosomes to recruit Gαi3 to the integrin signaling complex. Expression of GIV in non-invasive cancer cells results in enhanced haptotaxis and invasion. Modulation of expression of integrins is an alternative mechanisms used by cancer cells to control migration, invasion and metastasis. Human telomerase reverse transcriptase (hTERT) expression and telomerase activation are observed in 90% of human malignancies. hTERT plays an important role in cancer invasion by enhancing β1 integrin to promote the invasion of gastric cancer (Hu et al., 2015). The collaboration between integrins and GFR also accelerate tumor cell mobility and invasion. Clinical and functional analyses showed that CD151 and α3β1 integrin were elevated in glioblastoma. Both synergized with EGF/EGFR to accelerate tumor cell motility and invasion (Zhou et al., 2015). β1 integrin/kindlin and EGFR complexes increase breast and lung cancer cell migration (Li et al., 2013; Williams and Coppolino, 2014; Guo et al., 2015). Fibronectin matrix mediates PDGFR-β association with α5β1 integrin in focal adhesions and regulates cell migration (Veevers-Lowe et al., 2011). HGF-mediated c-Met activation induces collective cancer cell invasion through β1 integrin trafficking (Mai et al., 2014). All these data suggest that β1 integrins and GFR share the same signaling pathways to modulate migration of cancer cells. In human colorectal cancer, downregulation of the aryl hydrocarbon receptor nuclear translocator (ARNT or HIF-1β) promoted cancer cell migration and invasion through the activation of the fibronectin/β1 integrin/FAK signaling axis. Chemotherapeutic drugs inhibited ARNT expression and promoted invasion of residual tumor cells (Huang et al., 2015). In head and neck squamous cell carcinoma, disappearance of caveolin-1 expression in primary tumors is predictive of high risk of metastasis and is of bad prognosis. α2β1 and α5β1 integrins, both of which are regulated by caveolin-1, are responsible for the acquisition of motile, invasive, evasive and metastatic traits of tumors (Jung et al., 2015). MiR targeting of integrins represents a new way to endogenously regulate their expression. By targeting directly kindlin-2, miR-200b silenced the kindling-2/β1 integrin/AKT regulatory axis that ultimately suppresses the invasiveness of esophageal squamous cancer cells. miR-25 acts as a tumor suppressor in prostate cancer by direct functional interaction with the 3'UTR regions of proinvasive αv and α6 integrins (Zhang et al., 2015a; Zoni et al., 2015).

The tumor cell dissemination to a particular metastatic niche is dependent on the integrin repertoire expressed at the surface of cancer cells, blood and lymph compartment, vasculature, stromal cells as well as the composition and organization of ECM. For instance, a hepatic microenvironment favors the expression of α2β1 and α5β1 integrins on colorectal cancer cells which prompted colorectal cancer metastases to settle in the liver (Pelillo et al., 2015). αvβ3, α2β1 and α4β1 integrins play a key role in bone metastasis as their ligands are normally expressed by the bone-associated cells (Esposito and Kang, 2014). Knock-out mice for α11β1 integrin, a stromal cell-specific receptor for fibrillar collagen overexpressed by carcinoma-associated fibroblasts (CAF), prevent the metastatic potential of lung adenocarcinoma cells to bone, kidney, or brain (Navab et al., 2015). Lymph node metastasis (LNM) is recognized in clinical medicine as of bad prognosis for HNSCC patient. α2β1, α3β1, α6β1 integrins were identified as specific receptors that mediate the interactions between tumor cells and laminin present in the lymphatic environment (Fennewald et al., 2012; Soares et al., 2015).

## Integrins and neoangiogenesis

The role of integrins in developmental and pathological angiogenesis has been largely described (Avraamides et al., 2008). As a leader, the αvβ3/β5 integrin was long considered as a primordial player in tumoral neo-angiogenesis and its specific antagonist cilengitide was the first to reach clinical trials as an anti-angiogenic compound (Stupp et al., 2010). Unfortunately, cilengitide failed to improve the overall survival of glioblastoma patients in a multicentric randomized phase III clinical trial (Stupp et al., 2014). The need to understand the fine molecular events supporting integrin biology and functions appears currently as a priority in the field (Atkinson et al., 2014). Recent data indicate that dosage and timing of αvβ3 integrin antagonism are critical to pro- or anti- angiogenesis effects (Robinson et al., 2009; Steri et al., 2014). Hence, proof of principle that low doses of cilengitide, which were shown to promote angiogenesis, may be used in a “vascular promotion therapy,” opens a new field in integrin antagonist usefulness (Wong et al., 2015). Integrin α5β1 was also highlighted as a pro-angiogenic driver with an increased expression in neo-angiogenic tumoral vessels (Schaffner et al., 2013). However, recent data using KO mice models challenged the implication of α5, αv and their matrix ligand fibronectin in the tumor angiogenesis (Murphy et al., 2015). Discrepancies observed between the effects of the gene deletions and those of integrin-matrix adhesion blocking compounds on angiogenesis led to the interesting hypothesis that the latter may induce some anti-angiogenic function in the integrins. A better understanding of the integrin signaling pathways will help to understand their fine tuning in endothelial cells. Recent data explored the molecular regulation of angiogenesis through β1 integrin activation/inhibition and revealed cross-talks between angiopoietin-2, Arf6, VE-cadherin or MAP4K4 and β1 pathway (Hakanpaa et al., 2015; Hongu et al., 2015; Vitorino et al., 2015; Yamamoto et al., 2015).

Integrins also participate to anti-angiogenic therapy resistance. One of the most studied anti-angiogenic therapy is Bevacizumab, a monoclonal antibody against VEGF-A. Addition of Bevacizumab to adjuvant therapies in multiple cancer types improved progression free survival of patients (Ahmadizar et al., 2015). In brain tumors, anti-VEGF therapy led to bevacizumab-resistant recurrent glioblastoma with two different phenotypes, one of which appeared as infiltrative and the other as proliferative (de Groot et al., 2010; DeLay et al., 2012). Interestingly, the former expressed more α5β1 integrin and fibronectine. β1 integrin targeting was shown to disrupt the resistance toward Bevacizumab (Carbonell et al., 2013; Jahangiri et al., 2014).

## Integrins and reprogramming of energy metabolism and the evasion of immune destruction

Unlike normal cells, tumor cells use aerobic glycolysis (the Warburg effect) rather than oxidative phosphorylation (OXPHOS) to generate energy. This reprogramming of glucose metabolism is promoted by Twist through a β1-integrin/FAK/PI3K/AKT/mTOR pathway (Yang et al., 2015). Interestingly, it was shown recently that aerobic glycolysis or OXPHOS deregulation may enhance cancer cell migration and invasion through modulation of β1 integrin pathway (Yang et al., 2014; Nunes et al., 2015). Concerning the immune system, αv integrin upregulation can promote ADCC (antibody-dependent-cell-mediated cytotoxicity) but also link drug resistance with immune evasion (Jinushi et al., 2012; Anikeeva et al., 2014). Local immune response can be abrogated by tenascin C/α5β1 integrin to promote metastasis (Jachetti et al., 2015). A phenomenom named “integrin transregulation” can enhance tumor immunity through an increase in T-cell entry into melanomas (Cantor et al., 2015). Innate immune cells can promote tumor metastasis in dedicated environment. Interestingly, it was proposed that immune cell-derived microparticles may transfer αMβ2 integrin to tumor cells leading to their migration *in vitro* and metastasis *in vivo* (Ma et al., 2013). This recent literature suggests that an exponential growth of data will be available in the future characterizing the roles of integrins in these two new hallmarks of cancer.

## Conclusions

**Figure 1:**
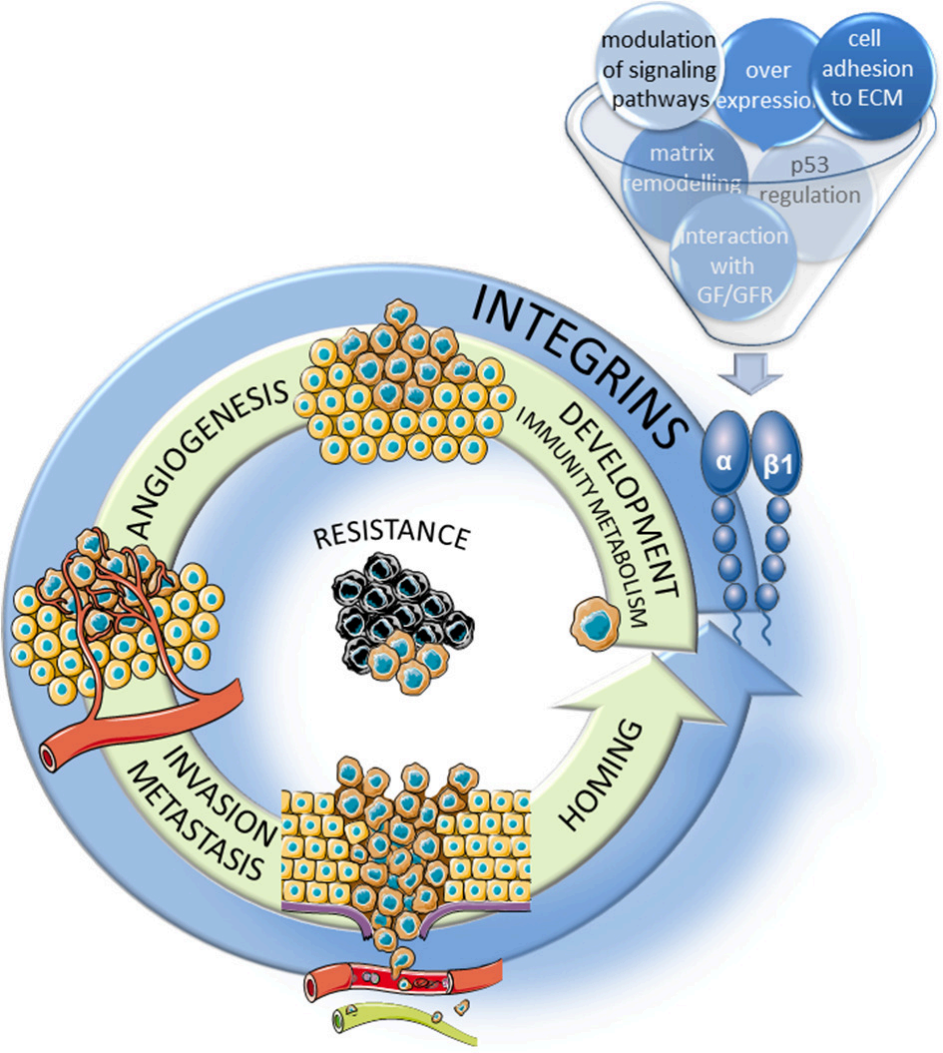
**Implication of β1 integrins in the hallmarks of cancer**. β1 integrins participate, through several mechanisms, to the major steps of tumor progression including the development of the tumor and of new vessels, migration/invasion into the surrounding stroma and extravasion through neoangiogenic vessels and homing in new tissues to form metastasis. In addition, these integrins participate largely to the resistance to therapies.

The goal of this review was to give a brief and non-exhaustive overview of the most recent data about the implication of β1 integrins in different hallmarks of cancer (Figure 1). Examples given here stress the complexity of the integrin signaling pathways which will largely depend on the tumor context under consideration. Micro environmental cues as well as molecular features of the tumoral cells themselves will determine which integrin(s) may be preferentially targeted. Increasing knowledge on how the integrin expression and functions are modulated is mandatory to propose associated therapies more susceptible to eradicate tumors.

## Author contributions

MD planned and edited the manuscript; AB, GR, SM, IL, and ML made the experiments leading to the laboratory publications cited in the review; AB, GR, ML, IL, SM, and MD wrote the manuscript.

### Conflict of interest statement

The authors declare that the research was conducted in the absence of any commercial or financial relationships that could be construed as a potential conflict of interest.
